# An Interference-Assisted Thermal Bonding Method for the Fabrication of Thermoplastic Microfluidic Devices

**DOI:** 10.3390/mi7110211

**Published:** 2016-11-22

**Authors:** Yao Gong, Jang Min Park, Jiseok Lim

**Affiliations:** School of Mechanical Engineering, Yeungnam University, Daehakro 280, Gyeongsan, 38541 Gyeongbuk, Korea; manyjoy321@gmail.com

**Keywords:** lab-on-a-chip, thermal bonding, interference fit, injection molding

## Abstract

Solutions for the bonding and sealing of micro-channels in the manufacturing process of microfluidic devices are limited; therefore, further technical developments are required to determine these solutions. In this study, a new bonding method for thermoplastic microfluidic devices was developed by combining an interference fit with a thermal treatment at low pressure. This involved a process of first injection molding thermoplastic substrates with a microchannel structure, and then performing bonding experiments at different bonding conditions. The results indicated the successful bonding of microchannels over a wide range of bonding pressures with the help of the interference fit. The study also determined additional advantages of the proposed bonding method by comparing the method with the conventional thermal bonding method.

## 1. Introduction

A microfluidic device is a miniaturized device in which micrometer-scale components, such as microchannels, micromixers, and microincubators, are integrated to achieve high-throughput analysis with respect to chemical and biomedical reactions [1,2,3,4,5,6]. Recently, the market for lab-on-a-chips utilizing microfluidic devices has rapidly increased with increase in the need for point-of-care systems. Therefore, this has led to the availability of various types of commercial microfluidic devices in the market [7].

Microfluidic devices can be fabricated from various materials including glass, silicon, and polymeric materials [8,9]. Poly-dimethylsiloxane (PDMS) is the most common material used for the fabrication of microfluidic devices in the laboratory, as it allows simple replication and sealing procedures. Furthermore, advantages of PDMS, such as biocompatibility, high gas permeability, and superior optical transmittance, are required in many cases for biomedical applications. However, the issue of a long fabrication time is a disadvantage of PDMS; thus, it is difficult to use PDMS as a material for several practical applications.

An extensive review of commercial microfluidic devices [7,10] suggested that thermoplastic materials are the most commonly used materials in practical applications of microfluidic devices. The main advantages of thermoplastic materials include low cost and disposability of the device due to mass production.

The manufacturing process of thermoplastic microfluidic devices consists of the following major steps: (i) fabrication of a microfeatured substrate; and (ii) bonding the substrates and interconnecting the substrates with external devices [11]. In contrast to the first step, the second step continues to present a challenge, and thereby necessitates further technical developments as noted by current research [12].

Previous studies proposed various methods, such as adhesive bonding, solvent assisted bonding, local welding, and thermal bonding, for the development of bonding solutions for thermoplastic-based microfluidic devices [12,13]. A comprehensive review on the various bonding method can be found in the recent review paper by Temiz et al. [12]. Each proposed bonding method exhibits its own set of advantages and disadvantages when compared with other methods.

For example, adhesive bonding is a simple method in which strong bonding can be achieved at room temperature. However, the surface properties of adhesive bonding can be modified. Additionally, an adhesive can clog a microchannel. In contrast, local welding offers a fairly strong degree of bonding without changes in the surface chemistry, but requires special equipment for precise welding.

**Figure 1:**
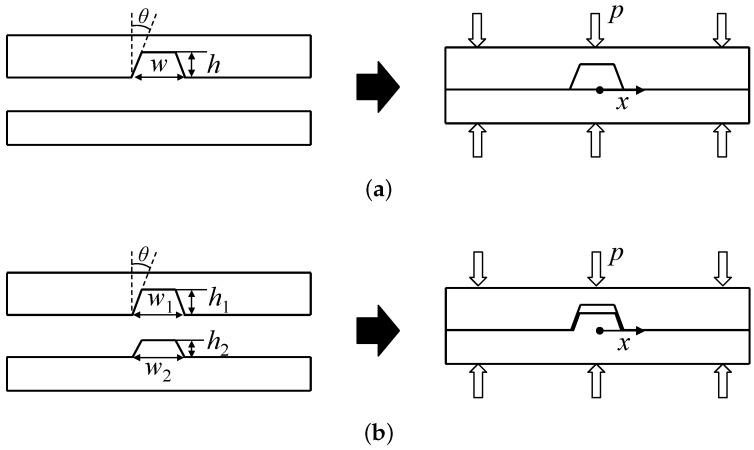
Schematics of (**a**) the conventional thermal bonding method; and (**b**) interference-assisted thermal bonding method.

A thermal bonding process can be considered an appropriate solution. In this process, two substrates are first heated to a temperature approximately equal to the corresponding glass transition temperatures of the respective substrates, and this is followed by the application of pressure (*p*). Molecular diffusion occurs across the contact surface between the two substrates during this process. A bonded product is finally obtained after the cooling process. Figure 1a shows the schematic of a conventional thermal bonding method.

The thermal bonding method offers certain advantages for microfluidic device assembly under optimized processing conditions. Specifically, it offers sufficiently high bonding strength, and the chemical properties of the thermoplastic surface remain unchanged [11,13,14]. However, the conventional thermal bonding method also entails issues limiting its application in practical manufacturing processes. In particular, it necessitates the uniform application of pressure over the entire substrate to seal the microchannels and other microfluidic components, and this is not trivial, especially in cases involving a large bonding area. Furthermore, given that the bonding area changes following the channel design, it is necessary to precisely calculate and control the applied force to avoid delamination or channel collapse during the bonding process [13].

As noted above, the most challenging part in the bonding of thermoplastic microfluidic device would be the uniformity of the applied pressure. In addition to this issue, warpage of the substrate is also a critical factor affecting the bonding uniformity [15]. To resolve these issues, Park et al. developed a pressure chamber system where pressure and temperature can be precisely controlled [16]. This method, however, still have limitations regarding the productivity. Since then, there has not been much progress made regarding the thermal bonding method.

This study involved the development of a new bonding method that combined interference fit and thermal bonding. This bonding method, termed the interference-assisted thermal bonding method, offered additional benefits when compared with the conventional thermal bonding method. In this method, the bonding performance was less sensitive to the applied pressure, since the bonding occurred due to the stress induced by mechanical interference between the substrates. The interference-assisted thermal bonding method for a simple microfluidic device was numerically analyzed and experimentally proved in the study.

## 2. Interference-Assisted Thermal Bonding Method

Figure 1b shows a schematic of the interference-assisted thermal bonding method. The upper substrate contains a microchannel of width $w_{1}$, while the lower one has a micro-rib of width $w_{2}$, and both the structures have a draft angle of θ. If $w_{2}-w_{1}>0$, then interference between the microchannel and the micro-rib exists. This interference causes a pressure higher than the applied pressure (denoted by *p*) to develop along the sidewall of the micro-rib. This leads to strong bonding along the sidewall, and thus the microfluidic system is sealed.

Typically, the bonding strength increases with the applied pressure in the thermal bonding of thermoplastics [14]. Numerical simulation was first performed prior to the experimental implementation of the proposed bonding method to quantitatively analyze the pressure distribution along the contact surface between the two substrates. The numerical simulation involves two-dimensional models as shown in Figure 1. The microchannel width ($w_{1}$) and height ($h_{1}$) were fixed to 500 μm and 700 μm, respectively. Other geometrical parameters of draft angle (θ), micro-rib width ($w_{2}$) and height ($h_{2}$) were varied to observe their effect on the pressure distribution. The width and thickness of the substrate were 5 mm and 1 mm, respectively. The process was assumed to be isothermal at 95 °C, and ANSYS software was used to only perform the structural analysis. An elastic modulus of 533 MPa and a Poisson ratio of 0.4 were applied with respect to the material properties of the substrates. A constant pressure was applied at the upper boundary of the upper substrate, while the lower boundary of the lower substrate was fixed. The left and right boundaries of the substrates were constrained such that they did not exhibit any displacement in the lateral direction, and a constant friction coefficient of 0.2 was applied for the contact condition between the two substrates.

**Figure 2:**
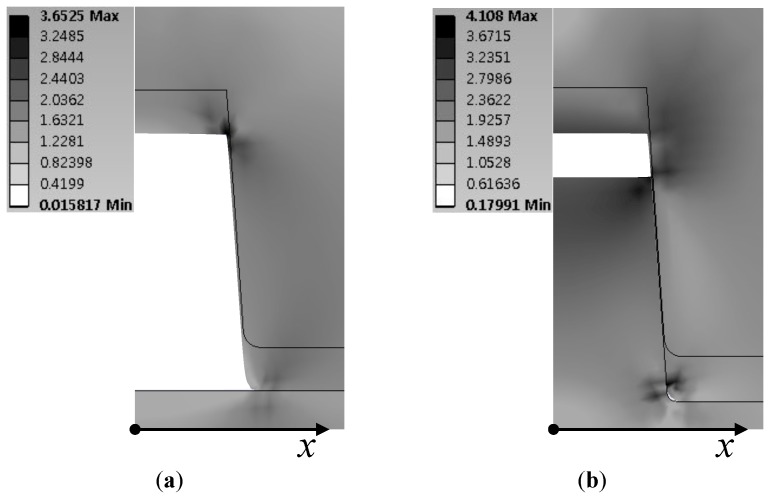
Simulation results of von Mises stress distribution around microchannel (**a**) without the interference fit and (**b**) with the interference fit. Applied pressure is 1 MPa. Interference height, width and draft angle are 500 μm, 10 μm and 4°, respectively.

Figure 2 shows the stress distribution around the microchannel when a pressure *p* of 1 MPa was applied, and the interference is 10 μm. The overall stress level remained almost the same with the interference, but there was a significant change in the distribution especially at the corner region of the microchannel.

**Figure 3:**
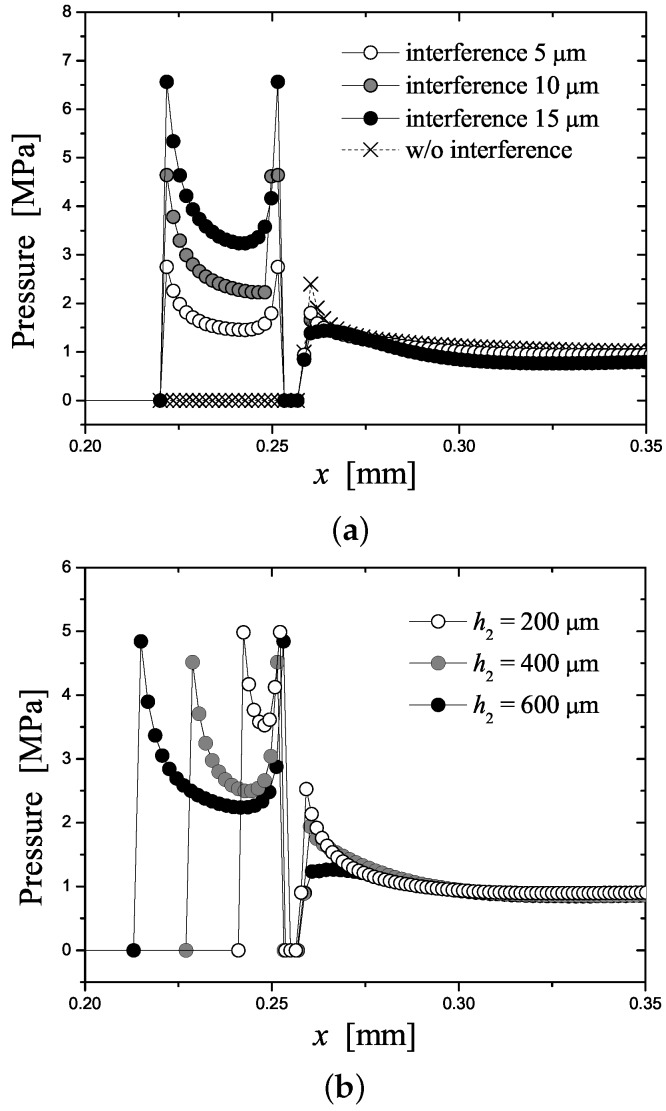

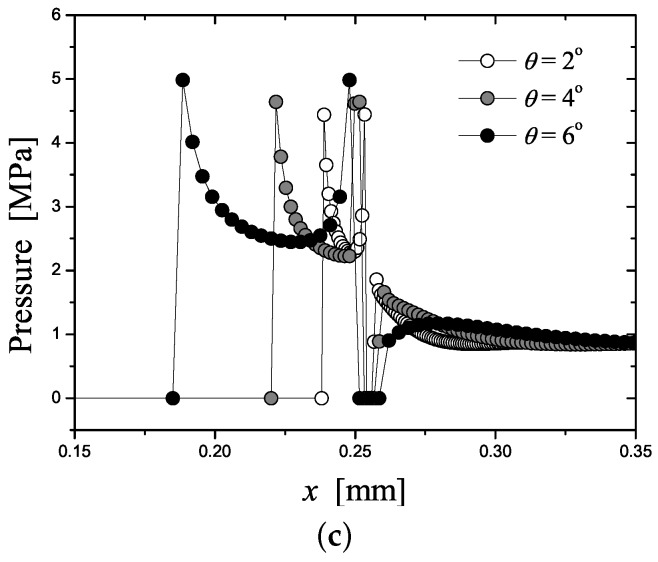
Simulation results of profiles of contact pressure between the two substrates: (**a**) Interference width is varied, while interference height ($h_{2}$) and draft angle (θ) are fixed as 500 μm and 4°; (**b**) Interference height is varied, while interferenc width and draft angle are fixed as 10 μm and 4°, respectively; (**c**) Draft angle is varied, while interference width and height are fixed as 10 μm and 500 μm, respectively. Applied pressure is 1 MPa.

Figure 3 provides further details as it shows the pressure distribution applied on the contact surface between the two substrates. In particular, effect of the interference width ($w_{2}-w_{1}$), interference height ($h_{2}$) and draft angle (θ) was investigated to provide design guideline of the interference fit. Shown in Figure 3a is the effect of the interference on the pressure distribution when $h_{2}=$ 500 μm and $\theta =4^{\circ }$. In the conventional case, the pressure was close to 1 MPa in the overall region (*x* > 0.27 mm) except for the corner region (0.25 mm < *x* < 0.27 mm) where the pressure was locally concentrated as expected. In the interference-assisted case, the pressure was slightly reduced in *x* > 0.26 mm, while it could be significantly increased along the interference region (0.22 mm < *x* < 0.252 mm). The tendency grew more pronounced as the interference increased. This numerical result suggested that increased bonding strength could be achieved along the interference region while the remaining region was only slightly affected. However, it should be noted that one needs to apply the interference width of at least 10 μm in order to have the contact pressure along the interference region more than two times the applied pressure.

Figure 3b shows the effect of $h_{2}$ on the pressure distribution when $w_{2}-w_{1}=$ 10 μm and $\theta =4^{\circ }$. The maximum pressure at the interference region was found to almost independent of $h_{2}$, while the average value is almost inversely proportional to $h_{2}$. Therefore, when $h_{2}$ is further increased as $h_{2}\geq 400$ μm, the average pressure at the interference region is only slightly affected by $h_{2}$. From the view point of the process optimization, it would be better if the contact pressure is less sensitive to the geometrical parameter of the interference. In this regard, it is recommended to select the interference height ($h_{2}$) as at least two times the final microchannel depth ($h_{1}-h_{2}$). In contrast, the draft angle θ had only minor effect on the pressure distribution as shown in Figure 3c where $w_{2}-w_{1}=$ 10 μm and $h_{2}=$ 500 μm were applied. The maximum and average pressure at the interference region is slightly decreased as θ is decreased.

According to the numerical simulation result, the interference height and draft angle had only minor effect on the pressure distribution in comparison to the interference width did. As a result, interference height and draft angle were fixed to 500 μm and $4^{\circ }$, respectively, in the following experiments. In order to determine appropriate value of the interference width, one would need more elaborate study than the simulation, since more complicated non-linear deformation and fracture phenomena could take place in practice, which were not included in the present simulation. Therefore, the interference width $w_{2}-w_{1}$ were determined based on experimental tests, which will be discussed in the next chapter.

## 3. Experiments

In the study, the injection molding process replicated two substrates, which were used for the bonding experiments. Mold inserts were fabricated by micro-machining, and the thermoplastic material poly(methyl metacrylate) (PMMA, IH830H, LG Chem.) was used in the injection molding process. Table 1 shows the injection molding condition. It should be noted that the injection molding cycle time required to obtain a substrate was only 27 s.

An in-house hot embossing equipment was used to perform the bonding experiments. The two substrates were first interference fitted and mounted on the hot embossing equipment. The specimen was then heated to the bonding temperature, and this was followed by the application of pressure for 3 min. The pressure was released after cooling the specimen to 50 °C, and the final product was obtained. In the study, the bonding temperature was varied between 95 °C, 97 °C and 100 °C, The bonding pressure was varied from 0.1 MPa to 3.0 MPa in order to observe the effect on the bonding quality. The processing condition is selected based on the previous work by Zhu et al. [14].

**Figure 4:**
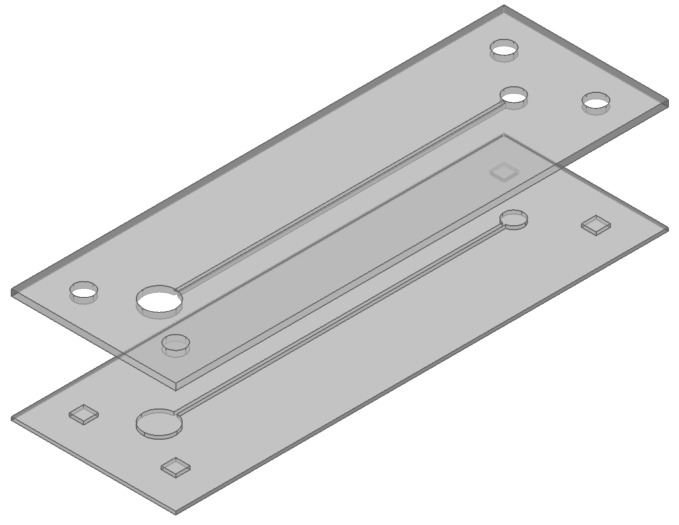
Schematics of the two substrates used in the bonding experiment. The upper substrate has two holes and a microchannel in the middle and four holes are located near the corner. The lower substrate has micro-rib and protrude structures which will result in interference fit with the upper substrate.

Figure 4 shows the schematics of the two substrates fabricated by the injection molding process. Each substrate had a thickness of 1 mm. With respect to the alignment between the two substrates, holes and rectangular pillars were located near the corners of the upper and lower substrates, respectively. These alignment structures of the holes and pillars also displayed interference fit in between the substrates. Each structure on the substrates had a draft angle of 4° to ease the demolding involved in the injection molding process. The upper substrate had two holes (with diameters of 3 mm and 5 mm) and a microchannel (with a width of 500 μm and a depth of 700 μm).The lower substrate exhibited protruded microstructures (with a height of 500 μm) with respect to the interference fit with the upper substrate. The lateral dimensions of the protruded microstructures were designed such that the interference was 10 μm, which was determined from the following experiments.

As discussed with Figure 3a, the interference width should be at least 10 μm to achieve significant increase of the contact pressure along the interference region. However, it should not be too large so that the interference does not deteriorate the original shape of the microfluidic system. In addition, it was necessary for the interference dimension to be within the resolution range of the manufacturing method employed to fabricate the mold inserts. In this study, mold inserts having different interference width of 10 μm to 200 μm were fabricated by micro-machining process, and bonding experiments were performed to determine the appropriate value of the interference. According to the test, specimens having the interference width larger than 20 μm had a fracture along the upper wall of the microchannel during the bonding process. This fracture was mainly due to a weldline of which details will be shown and explained in the next chapter. As a result, the interference width of 10 μm was applied in this study.

## 4. Results

**Figure 5:**
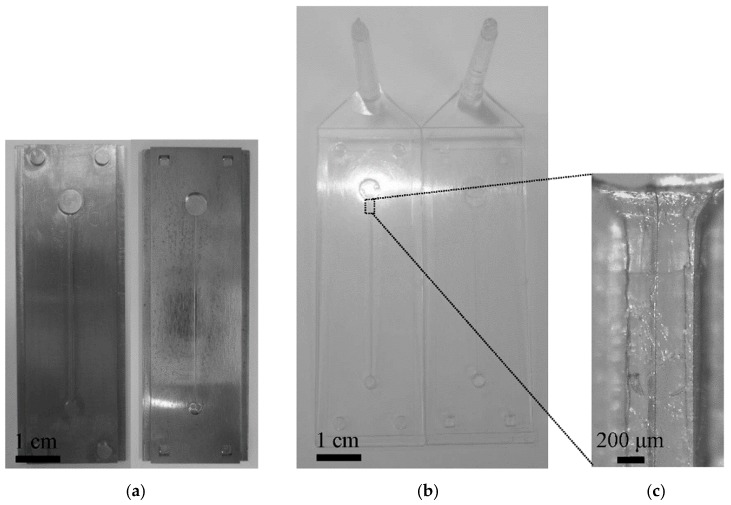
Photographs of mold inserts (**a**), injection molded substrates (**b**) and a micrograph of the microchannel (**c**). There is weldline along the center of the microchannel in the micrograph.

Figure 5 shows photographs of the mold inserts, injection molded substrates and a micrograph of the microchannel. Several types of methods were available for the fabrication of the mold inserts [17]. In the case used in the present study, the minimum feature size of the microchannel was 500 μm, and the maximum aspect ratio was 1.4. The study involved obtaining an interference of 10 μm, and thus the feature tolerance of the manufacturing method could correspond to an order of micrometers. Additionally, fine roughness of the sidewalls was not required. Micro-machining satisfied these requirements and could provide suitable mold inserts. It might be mentioned that one should use other fabrication methods for the mold insert to produce devices having smaller features.

A fan gate was used to obtain uniform filling of the substrate. With respect to the injection molding of the substrate with holes and a microchannel (as shown in Figure 5), a weldline developed along the upper wall of the microchannel due to the presence of a hole. This weldline can be observed clearly in the magnified view of the microchannel in Figure 5. The weldline could result in a fracture of the upper wall during the interference-assisted thermal bonding process because of the weak mechanical properties of the welding. A mold temperature higher than that in the conventional molding condition of PMMA material was applied to avoid such defects (Table 1).

**Figure 6:**
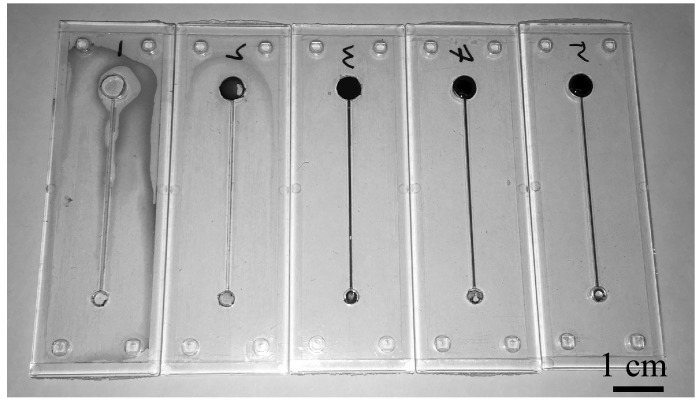
Sealing test results of products bonded at different bonding pressures of 0.1 MPa, 0.5 MPa, 1.0 MPa, 1.5 MPa, and 2.0 MPa (from the left to right). The bonding temperature is 95 °C. Dyed water is injected through the upper chamber to visualize the sealing.

Figure 6 shows five different products obtained from bonding experiments with different bonding pressures. A dyed liquid was introduced at the larger well to confirm the sealing of the microchannel and wells. The products bonded at a pressure of 1 MPa and products bonded at higher pressure showed a distinct microchannel and two wells, thereby indicate complete sealing. Conversely, products bonded at low pressures of 0.1 MPa and 0.5 MPa exhibited leakage due to insufficient bonding.

**Figure 7:**
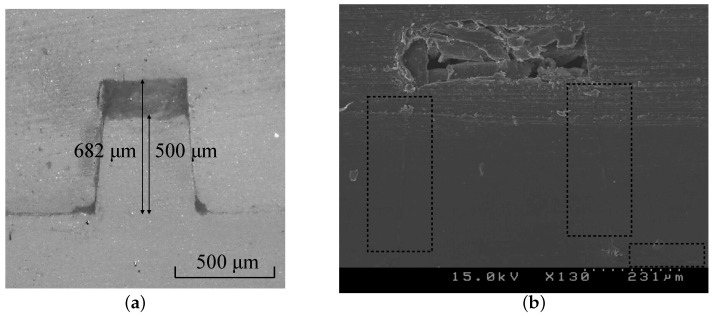
Cross-sectional micrographs of the microchannel observed by (**a**) optical microscope; and (**b**) scanning electron microscope (SEM). The dotted boxes in the SEM micrograph indicate the contact region. The bonding temperature and bonding pressure are 95 °C and 2 MPa, respectively.

Cross-sections of the bonded products were observed to investigate the effect of the bonding condition on the final shape of the microchannel. Figure 7 shows the product bonded at 2.0 MPa and 95 °C. The micro-rib was retained securely without significant deformation. The microchannel only exhibited a slight deformation, and its depth was 682 μm. Figure 7b presents the scanning electron microscope (SEM) micrograph to confirm the bonding along the contact region. The other products that bonded at lower pressures of 1.0 MPa and 1.5 MPa also displayed approximately the same cross-sectional structures as shown in Figure 7 (although this was not shown in this study). However, the only notable difference was that the bonding in the plane became weaker as the pressure decreased.

**Figure 8:**
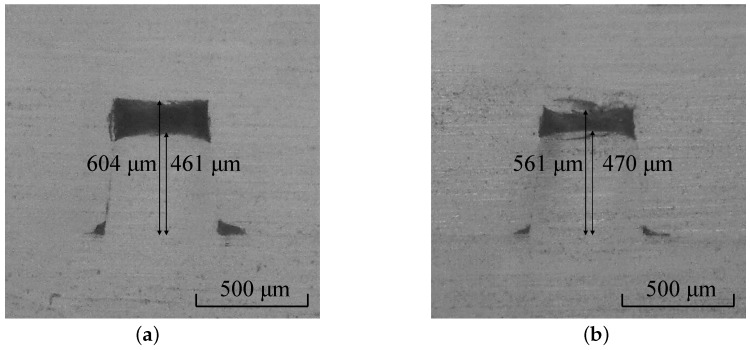
Cross-sectional micrographs of the microchannels bonded at temperatures of (**a**) 97 °C; and (**b**) 100 °C. The bonding pressure corresponds to 2 MPa.

Figure 8a,b show the cross-sections of the products bonded at higher temperatures of 97 °C and 100 °C, respectively. The results indicated that the microchannel collapsed further as the temperature increased. Additionally, the micro-rib was deformed when compared to that shown in Figure 7a. However, the height did not monotonically decrease with the temperature. This was attributed to the squeezing effect in the lateral direction, and this squeezing effect was reflected in the curved profile of the top surface.

## 5. Discussion and Conclusions

This study proposed a new bonding method that combined a thermal bonding method and an interference fit. The performance of the proposed method was experimentally investigated. This section discusses several features of the proposed bonding method and particularly focuses on the comparison of the proposed method with the conventional thermal bonding method.

In the present bonding method, the uniformity of the applied pressure did not affect the sealing of the microfluidic system to the same extent as that in the conventional thermal bonding method. The microfluidic system could be sealed first because of the interference fit between the microchannel and the micro-rib despite the potential lack of uniformity in the applied pressure over the entire substrate. This feature provides a substantial advantage. This is especially significant in cases where the substrate size increases such that the application of uniform pressure is more difficult to achieve.

In the proposed method, it is not necessary for the entire substrate to be completely bonded. It is only necessary for the sidewall of the microfluidic system to be strongly bonded by using the interference fit. This feature offers advantages in terms of process optimization and productivity. For example, as shown in Figure 6, it is possible to obtain well-sealed products with a bonding pressure ranging from 1 MPa to 2 MPa. This corresponded to a relatively wider processing window when compared with that of the conventional thermal bonding method. This indicated that process optimization was easier in the proposed method. Additionally, the proposed method reduced the probabilities of seal defects in the final product.

However, the proposed method also had certain disadvantages. First, the proposed method necessitates an additional mold insert, and this increases the overall manufacturing cost. In addition, the micro features should have extra height for the interference, which makes the mold insert fabrication more difficult. Also a possibility of bends or warpages with respect to a substrate after bonding was associated with the proposed method because of the presence of the interference. This was resolved in this study by introducing alignment structures near the corner of the substrate. As discussed previously, these alignment structures also displayed interference-assisted bonding, which prevented the bend or warpage of the substrate. Such structures could be introduced based on the design of the microfluidic system.

Despite these drawbacks, the interference-assisted thermal bonding method evidently offers certain advantages over the conventional thermal bonding method, especially with respect to productivity. Hence, it is expected that the proposed method will be widely used in practical manufacturing processes.

## Figures and Tables

**Table 1:** Injection molding condition.

Parameter	Value
Mold temperature	85 °C
Nozzle temperature	230 °C
Packing pressure	65 MPa
Packing time	3 s
Injection time	0.5 s
Cooling time	10 s
